# Effect of grazing on methane uptake from Eurasian steppe of China

**DOI:** 10.1186/s12898-018-0168-x

**Published:** 2018-03-20

**Authors:** Shiming Tang, Yujuan Zhang, Xiajie Zhai, Andreas Wilkes, Chengjie Wang, Kun Wang

**Affiliations:** 1 Department of Grassland Science, China Agricultural University, Beijing, 100193 China; 2 Institute of Grassland Science, Chinese Academy of Agricultural Science, Hohhot, 010010 China; 3 Values for Development Limited, Bury St Edmunds, IP33 3EQ UK; 4 College of Ecology and Environmental Science, Inner Mongolia Agricultural University, Hohhot, 010018 China

**Keywords:** Grazing, Steppe, CH_4_ uptake, Soil, Meta-analysis, China

## Abstract

**Background:**

The effects of grazing on soil methane (CH_4_) uptake in steppe ecosystems are important for understanding carbon sequestration and cycling because the role of grassland soil for CH_4_ uptake can have major impacts at the global level. Here, a meta-analysis of 27 individual studies was carried out to assess the response patterns of soil CH_4_ uptake to grazing in steppe ecosystems of China. The weighted log response ratio was used to assess the effect size.

**Results:**

We found that heavy grazing significantly depressed soil CH_4_ uptake by 36.47%, but light and moderate grazing had no significant effects in grassland ecosystem. The response of grassland soil CH_4_ uptake to grazing also was found to depend upon grazing intensity, grazing duration and climatic types. The increase in soil temperature and reduced aboveground biomass and soil moisture induced by heavy grazing may be the major regulators of the soil CH_4_ uptake.

**Conclusions:**

These findings imply that grazing effects on soil CH_4_ uptake are highly context-specific and that grazing in different grasslands might be managed differently to help mitigate greenhouse gas emissions.

**Electronic supplementary material:**

The online version of this article (10.1186/s12898-018-0168-x) contains supplementary material, which is available to authorized users.

## Background

Methane (CH_4_) is a long-lived greenhouse gas (average atmospheric residence about 7.9 years) [1], contributing approximately 30% of total net anthropogenic radiative forcing, which is second only to the radiative forcing of carbon dioxide (CO_2_) [2]. The concentration of atmospheric CH_4_ has been increasing because of anthropogenic activities over the last 150 years [3], reaching 1813 ppb in 2011, which is 159% higher than the pre-industrial level [4]. These changes can exert strong effects on terrestrial carbon cycles and global warming. Natural soils are the second largest sink of atmospheric CH_4_ after oxidation in the troposphere by OH radicals, with an estimated global sink of 20–45 Tg CH_4_ year^−1^ [2]. Grassland soils play a considerable role in mitigating greenhouse gas emissions because grasslands are one of the largest terrestrial biomes worldwide [4, 5]. Most studies of CH_4_ uptake have been conducted in grasslands of North America [6–9] and Europe [10–12]. The Eurasian steppe has only received attention in recent years [13–18]. In the Eurasian steppes, unprecedented increase in grazing pressure has led to severe grassland degradation, which in turn decreases CH_4_ uptake of soil [17, 19, 20].

China’s grasslands are representative of the Eurasian steppe in terms of climate, topography, soils properties, vegetation composition and land use history [21]. Generally, China’s steppe ecosystems are classified into desert steppes, typical steppes, meadow steppes and alpine steppes [22], and better understanding of soil-atmosphere CH_4_ exchange dynamics in this region can contribute to broader understanding of such dynamics in arid and semiarid areas. As elsewhere in the Eurasian steppe, grazing is the main land use, and most grasslands have been grazed for several decades to centuries. There is clear evidence that grazing in grassland ecosystems alters the activity or community composition of soil microorganisms and vegetation in ways that lead to decreased soil CH_4_ uptake [19, 20]. Therefore, grazing may alter the loading of CH_4_ flux to the atmosphere, which may contribute to further global warming. Understanding how grazing management affects the CH_4_ budget is thus important, from both scientific and political perspectives.

The potential impacts of grazing on the soil CH_4_ uptake have been investigated in an increasing number of field experiment studies across the worldwide grasslands. In order to improve understanding of the responses of CH_4_ uptake of steppe soils to grazing, several studies have been conducted in China [14–17, 19, 23]. Previous studies have reported inconsistent grazing effects on soil CH_4_ uptake by grasslands [14, 24, 25]. Liu et al. [23] suggested that winter grazing decreased soil CH_4_ uptake during the growing season by 47% in a temperate semiarid steppes. Qi et al. [24] reported that continuous grazing promoted CH_4_ uptake during growing season in grassland. Contradictory responses of CH_4_ uptake to grazing may depend on differences in grazing intensities, grazing duration or soil environmental conditions. Therefore, there are still many uncertainties in the CH_4_ uptake responses of steppe soils to increased grazing pressure.

Most previous studies on soil-atmospheric CH_4_ exchange in grazed Eurasian steppes were restricted to a single grassland type [16, 18, 20], or a single grazing intensity in the growing season [17, 23] or spring-thaw period [18]. Few reports are available on soil CH_4_ uptake in the different grazing intensity and grazing duration [17]. Incomplete considerations of these difference may be increase uncertainty when the overall contribution of grassland ecosystems to the greenhouse effect is assessed [17]. More complete assessments contribute to better understanding of atmospheric CH_4_ uptake in grazed steppe, and help to identify effective measures to increase the effects of the terrestrial CH_4_ sink. Therefore, it is needed to compile the available data to reveal the underlying mechanisms of soil CH_4_ uptake responses to grazing.

To reveal general response patterns of CH_4_ uptake by steppe soil under grazing, we incorporated factors such as grazing intensity (light, moderate and heavy grazing), grazing duration (< 5 years, 5–10 years and ≥ 10 years), and climatic type (humid/semi-humid, ≥ 400 mm precipitation; arid/semi-arid, < 400 mm precipitation) using data from published papers reporting field experiments conducted in China’s steppes (Additional file 1: Note S1).

## Results

**Fig. 1:**
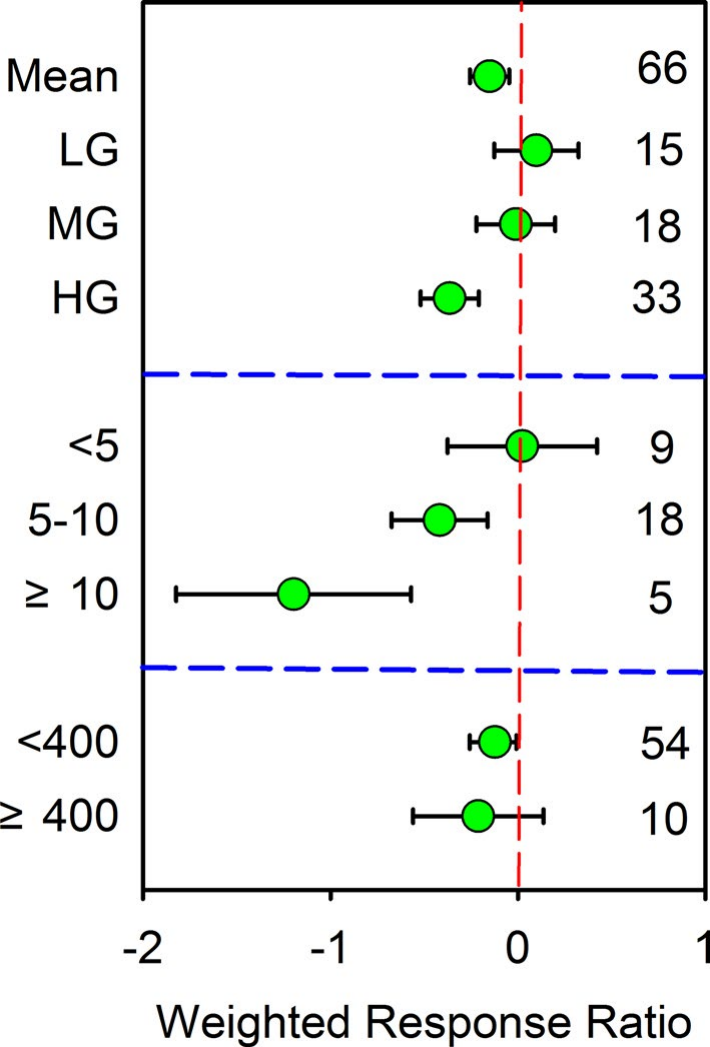
Weighted response ratio (RR ++) of CH_4_ uptake at different grazing intensities, in different steppe types, and grazing duration (years). Bars represent mean RR++ ± 95% confidence interval. The number of observations for each category used in the analysis is given in the figure. LG, MG, and HG are light grazing, moderate grazing, and heavy grazing, respectively

**Fig. 2:**
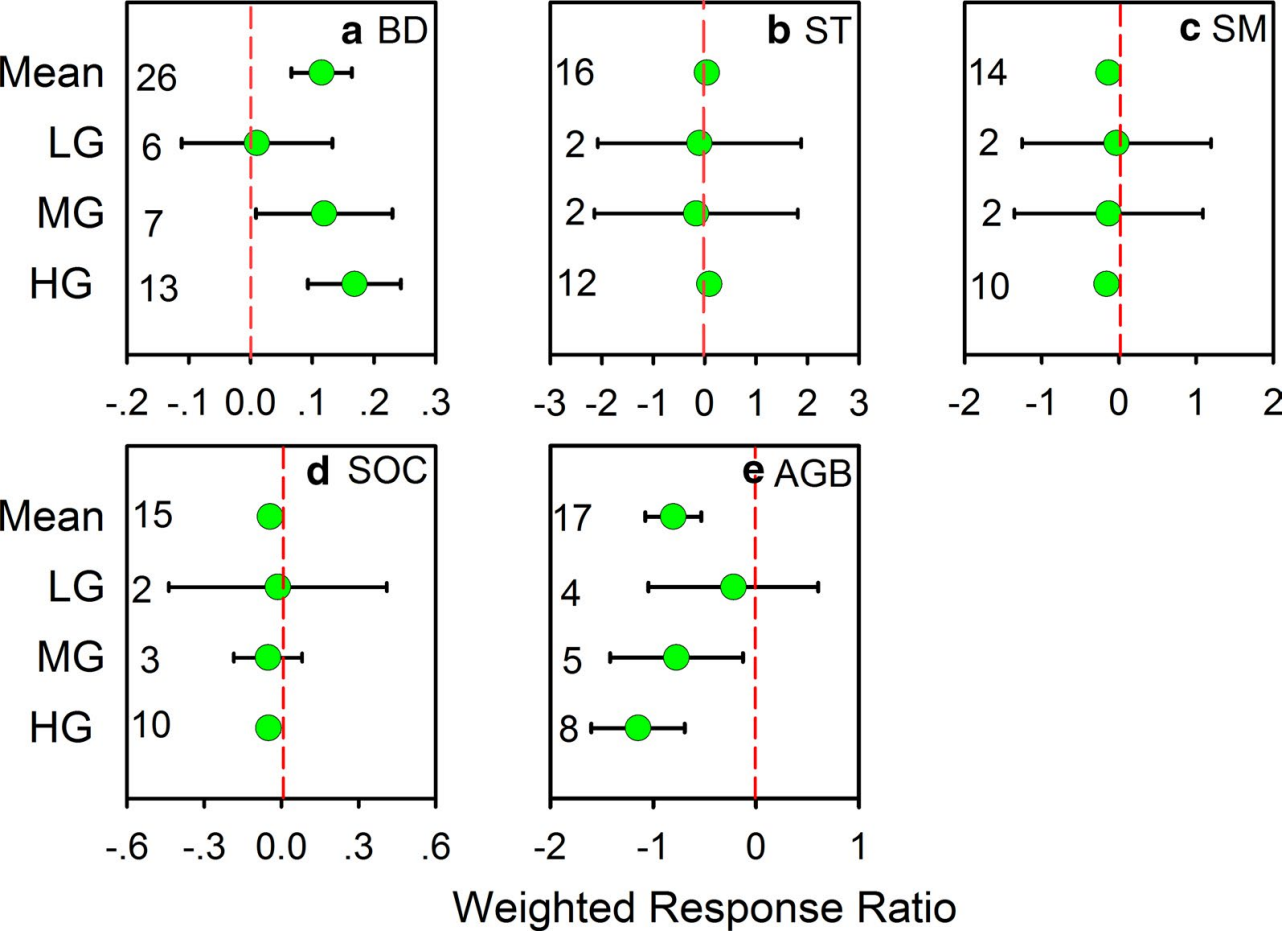
Effects of grazing on **a** soil bulk density (BD), **b** soil temperature (ST), **c** soil moisture (SM), **d** soil organic carbon (SOC) and **e** aboveground biomass (AGB), represented by weighted response ratio (RR ++) in different intensity. The dashed vertical lines were drawn at RR = 0. The bars represent 95% confidence interval. The number of observations for each category used in the analysis is given at each bar. LG, MG, and HG are light grazing, moderate grazing, and heavy grazing, respectively

Grazing effects showed a strong dependence on grazing intensity (Fig. 1, Table 1). The data suggest that soil CH_4_ uptake decrease as the grazing intensity increases, however, the effect is significant only under heavy grazing (− 36.47%, p < 0.05) (Fig. 1). The effects of light grazing and moderate on soil CH_4_ uptake were not significantly different (LG 9.77%; MG 1.22%) (Fig. 1). In addition, heavy grazing significantly reduced soil organic carbon by 5.01%, soil moisture by 16.09% (p < 0.05) and aboveground biomass by 114.83% but increased soil bulk density by 16.84% (p < 0.05), and soil temperature were not significantly different (8.81%, p > 0.5) (Fig. 2a–e).

**Table 1:** Effects of the independent variables on the response ratios, using between-group heterogeneity (Q_b_) of the CH_4_ flux response to grazing

Types	Categories	Q_b_	p
Grazing intensity	Light, moderate, heavy	15.43	0.002
Climatic type	< 400 mm, ≥ 400 mm	0.28	0.554
Grazing duration	< 5, 5–10, ≥ 10	18.42	0.007

**Fig. 3:**
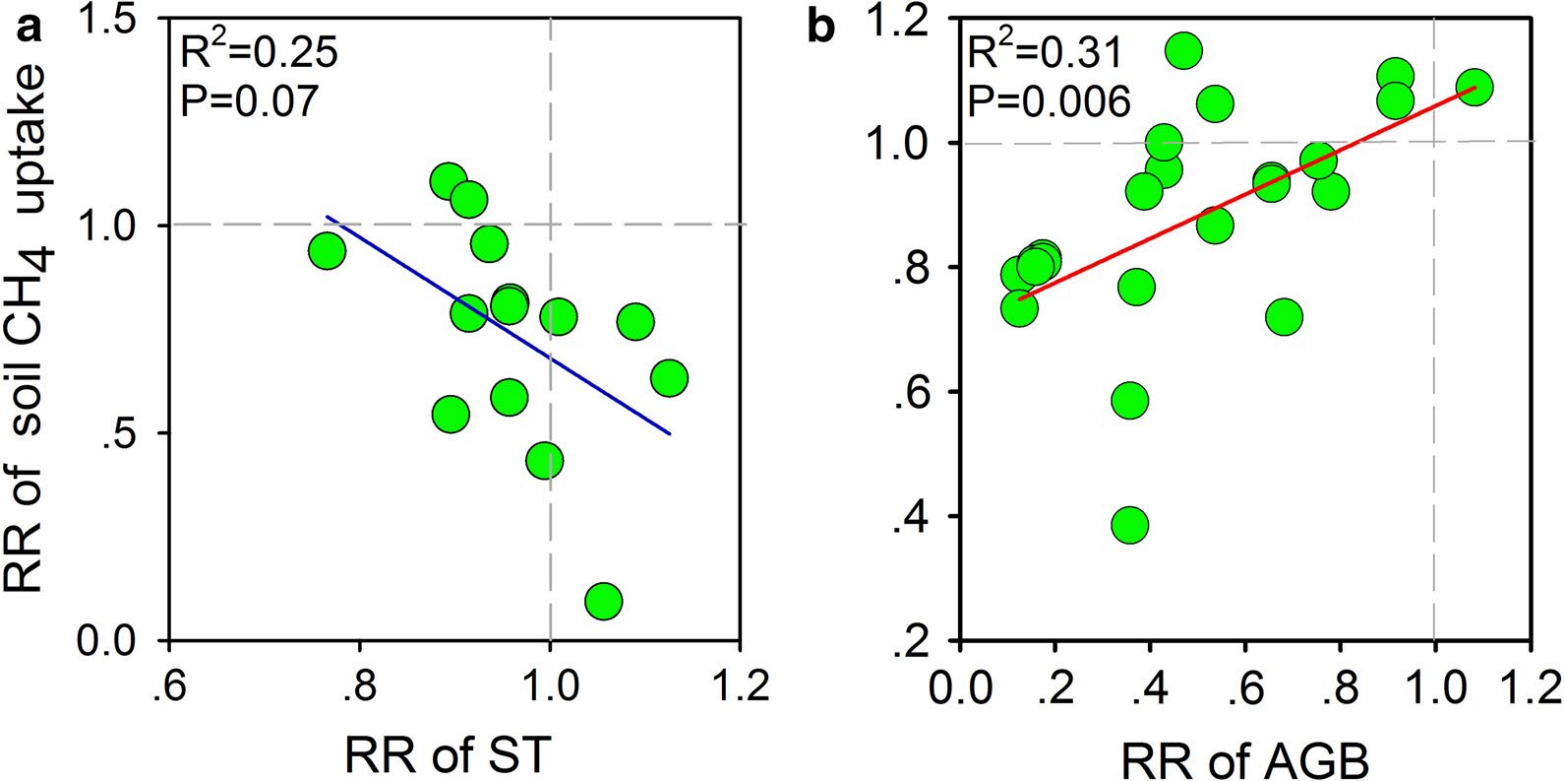
Relationships of response ratios (RR) of soil CH_4_ uptake flux with RR of **a** soil temperature (ST) and **b** aboveground biomass (AGB)

Grazing also significantly reduced soil CH_4_ uptake with the different grazing duration (p < 0.05). Significant difference in the soil CH_4_ uptake response was found between these categories (Q_b_ = 18.42, p = 0.007; Table 1). There is a strong trend indicating that CH_4_ uptake decreases with increasing duration of grazing activities, and that significant decreases occur when the grazing exceeds 5 years, and especially when it exceeds 10 years duration (Fig. 1). Averaging across studies in different climatic types, grazing significantly decreased soil CH_4_ uptake by 12.40% in precipitation < 400 mm (p < 0.01) (Table 1), but the effects of grazing on soil CH_4_ uptake were not significant in precipitation ≥ 400 mm (Fig. 1). In addition, our meta-analysis also showed that aboveground biomass displayed significant correlations with response ratio (RR) of soil CH_4_ uptake (Fig. 3b). The RR of soil CH_4_ uptake showed a trend of negative correlation with RR of soil temperature (Fig. 3a), but significantly positive correlation with RR of aboveground biomass was observed (Fig. 3b).

## Discussion

The atmospheric concentration of CH_4_ has dramatically increased since pre-industrial times because of human activities [2]. Grazing is an important disturbance to grasslands, which could have either positive or negative effects on the consumption of CH_4_ in grassland ecosystems [17, 19, 23]. Long-term overgrazing is the main cause of grassland degradation and associated dust storms in Eurasian grasslands. About 90% of grasslands in China are degraded to some extent, mainly due to overgrazing [22]. High-intensity ruminant grazing shifts net CH_4_ exchange in grassland ecosystem. In this study, grazing experiments with duration of longer than 5 years had a significant effect on soil CH_4_ uptake, while the experiments with duration of less than 5 years were not observed to pose an impact on soil CH_4_ uptake (Fig. 1). It likely also suggests that the grazing intensity is increased induced by grazing treatment in long term. The results of our study indicate that overgrazing has significant negative effects on the CH_4_ uptake of grassland soils in China, which would most likely cause a large decrease in soil CH_4_ uptake and a decrease in carbon sequestration of grassland in China.

This meta-analysis included a relatively small number of studies (n = 27) compared to other meta-analyses, and we were limited to considering interactions among factors with a wide range of values across grassland sites in China. In this study, we did not consider the grazing experiment at different grassland types because few reports were concentrated on the effects of grazing on soil CH_4_ uptake across different grassland types, and most of the identified studies were carried out in Inner Mongolia. The paucity of data from different grassland types indicates that insufficient research has been conducted on the impact of grazing on CH_4_ flux of grassland soils in China. However, we found soil CH_4_ uptake has different response to grazing at different precipitation types. The differential responses among climatic types estimated in our study suggests that arid and semi-arid region are more fragile to grazing practice in grassland ecosystems. Moreover, the vegetation cover also changed completely with different precipitation climatic type. It was found that vegetation influences methane uptake of soils indirectly via possible changes in methanotrophic communities due to changes of plant species [25]. Further research is required on the effects of grazing practice on CH_4_ fluxes of different grassland ecosystems in China.

Research indicates that light grazing may change the microbial community in grassland soils, which could result in a positive response of CH_4_ to light grazing [20, 26]. The results of this meta-analysis suggest light grazing has a 10.49% higher CH_4_ uptake than un-grazing, although this was not statistically significant (Fig. 1). Chen et al. [20] showed that light-to-moderate grazing with stocking rates of < 1 sheep ha^−1^ year^−1^ did not significantly change the annual CH_4_ uptake. However, heavy grazing reduced 24–31% of annual CH_4_ uptake in typical grassland of China. Tang et al. [17] also suggested in their study that grazing affected CH_4_ uptake fluxes variably in three grassland types (meadow grassland, typical grassland and desert grassland) of Inner Mongolia. Therefore, how CH_4_ flux responds to grazing is complex and should be studied carefully under different grassland type and conditions.

In this meta-analysis, we found that an increase in grazing intensity induced a reduction in CH_4_ uptake by grassland soils. Despite some limitations, the analysis revealed several mechanisms that may have contributed to a significant reduction in soil CH_4_ flux under high-intensity grazing. First, trampling by grazing animals compacts the topsoil and increases soil bulk density (Fig. 2a), which would decrease the diffusion of CH_4_ from the atmosphere into the soil [20, 23]. This may also reduce the amount of atmospheric O_2_ diffusing into the soil, which could result in an increase in anaerobic conditions and hence an increase in CH_4_ production. Second, soil organic carbon (SOC) decreased along the grazing gradient in this meta-analysis (Fig. 2d), which may result in a reduction of CH_4_ uptake in soil due to reduced soil carbon cycles in heavily grazed sites. Soils contain a large stock of SOC, and slight changes in SOC stock can represent large CO_2_ and CH_4_ fluxes [27]. Third, heavy grazing significantly reduced the aboveground biomass, which will decline soil water content (Fig. 2c, e) and increase water stress that could inhibit the activities of methanotrophs [20, 23] and further affect the net CH_4_ flux. In this study, RR of soil CH_4_ uptake increased linearly with the increase in RR of aboveground biomass but decreased linearly with RR of soil temperature (Fig. 3a, b). The decrease in aboveground biomass may reduce the soil moisture due to its effect on increasing evaporation [20]. However, more studies should be carried out in different grassland ecosystems to understand how soil CH_4_ uptake respond to soil water stress under different grazing intensity.

Urine and dung patches in grazed grassland are hotspots of CH_4_ emission [28, 29]. The higher potential for excreta induced CH_4_ emissions in grazed sites could offset some of the CH_4_ uptake by soils [20, 28]. In addition, as has been shown in other studies from a typical steppe in Inner Mongolia, grazing animals change the structure of the methane-oxidizing bacterial community. Zhou et al. [26] have reported that the community composition of soil methane-oxidizing bacteria was different between grazed and non-grazed sites. However, few studies reported other potentially important factors impacting CH_4_ fluxes, such as urine and dung patches, or soil microbial community under grazing management, so our analysis was not able to evaluate the effect of these factors on the responses of CH_4_ flux to grazing. These factors may be important because other studies in temperate grasslands of North America and China [9, 17, 20, 23] have suggested that CH_4_ flux may be associated with livestock urine and dung patches and soil methane-oxidizing bacteria.

Given the small total sample size, the inclusion of studies from that region of China may have some unclearly influenced the analysis of relationships with environmental variables. Our meta-analysis was limited by the relative paucity of published field studies, incomplete representation of different grassland types, and limited reporting of potentially important variables describing soil and vegetation properties. Filling these data gaps through field experiments and publication will be necessary to provide a stronger empirical basis for future large-scale assessments.

## Conclusions

Grazing has the potential to change soil CH_4_ uptake in steppe ecosystems, with consequent impacts on the carbon cycle and climate change. Our results and previous findings [14, 17, 20, 23] indicate that heavy grazing decreases soil CH_4_ uptake in steppe ecosystems in China. These findings in this and previous studies [17, 18, 20, 23] imply that grazing effects on soil CH_4_ uptake are highly context-specific and that grazing in different grasslands might be managed differently to help mitigate greenhouse gas emissions, especially when different grazing intensities are taken into consideration.

## Methods

### Data compilation

In order to identify all relevant studies on the effect of grazing on soil-atmosphere CH_4_ fluxes in China, a comprehensive search was conducted on the Web of Science and the Chinese magazine network (CNKI) database (before 2015). The search terms were ‘methane’ and‘flux’, ‘uptake’, ‘oxidation’, or ‘consumption’ and ‘grazing’. These searches resulted in over 27 papers that studied soil CH_4_ dynamics under grazing in steppes of China (Additional file 1: Note S1, Additional file 2: Table S1) by including studies that compared soil CH_4_ fluxes for grazers plot (different grazing intensity) compared to a paired un-grazed plot. In addition, we collected other variables of the treatment and control plots if reported, such as soil bulk density, soil organic carbon, soil moisture, soil temperature and aboveground biomass values. For CH_4_ flux, the preferred unit is flux per unit area per day (mg m^−2^ day^−1^), and all other flux units (e.g. µg m^−2^ h^−1^) were converted if data on plot area was provided in the paper. Mean values for CH_4_ fluxes were taken directly from the available literature. Data from graphs were extracted by digitizing the figures using a graph data extractor software (Graph Data Extractor by Dr. A J Matthews).

The data were selected according to the following criteria: (1) the studies reported changes in soil-atmosphere CH_4_ exchange in both grazing and control groups; (2) the means, standard deviations (SD) or standard errors (SE), and sample sizes (n) of the CH_4_ fluxes were provided or could be calculated from the studies; (3) relevant experimental information was reported, including grazing intensity, steppe type, grazing duration, mean annual precipitation (MAP) and mean annual temperature (MAT).

Due to large variation in both the types of steppe (i.e., desert, typical steppe, meadow and alpine steppe) and the grazing units (i.e., dry sheep equivalent ha^−1^, ha steer^−1^, animal unit month ha^−1^) reported in each study, we characterize grazing intensity based on the individual study authors’ own qualitative classification of grazing intensity as ‘light’, ‘moderate’, or ‘heavy’. If the cases were not given a qualitative grazing level, we classified data based on the authors’ qualitative description of the site. The stocking rates are given in more detail in Additional file 2: Table S1 according to the original studies. Sheep was the main grazing animal at most of studies.

### Meta-analysis

Following the techniques reported in Wan et al. [30] to calculate the response ratio (RR) of response variables to grazing, the meta-analysis was conducted using MetaWin 2.1 software package (Sinauer Associates, Inc., Sunderland, MA, USA) [31]. The natural log-transformed ratio of response variables in grazed (Xe) to un-grazed (Xc) plots was used to estimate the effect size of the grazing treatment. The means, standard deviations of response variables and sample sizes were reported or could be calculated. SE and confidence interval (CI) were transformed to SD before calculation. For studies that did not report SE, SD was assigned as 1/10 of mean [30, 32]. The weighted response ratio (ln RR) was calculated using MetaWin 2.1. The CI on effect-size estimates was generated by bootstrapping the data. The homogeneity test was used to further examine whether different groups of independent variables would result in different responses. The total heterogeneity (Q_T_) was partitioned into two components, within-group heterogeneity (Q_w_) and between-group heterogeneity (Q_b_). The Q statistic approximately followed a χ^2^ distribution, which allowed a significance test of the null hypothesis that all response ratios are equal [30, 33]. If the value of Q_b_ is larger than a critical value, this indicates that an independent variable had a significant impact on the response ratio [33]. Grazing was considered to have a significant effect on variables if the 95% CI did not overlap zero, whereas the grazing effects of different groups were considered to significantly differ from each other if their bootstrap CIs did not overlap [30, 33]. In addition, the relationships between change in CH_4_ flux and environmental factors were examined using correlation analysis. The significance of differences was assessed at p < 0.05 level.

## Additional files

**Additional file 1: Note S1.** List of studies used in the meta-analysis.

**Additional file 2: Table S1.** Summary of all experiments included in the meta-analysis in grassland of China.

## Abbreviations

CH_4_: methane
CO_2_: carbon dioxide
MAP: mean annual precipitation
MAT: mean annual temperature
RR: response ratio
ln(RR): weighted response ratio
SOC: soil organic carbon
SD: standard deviations
SE: standard errors
CI: confidence interval
Q_T_: total heterogeneity
Q_w_: within-group heterogeneity
Q_b_: between-group heterogeneity
UG: un-grazing
LG: light grazing
MG: moderate grazing
HG: heavy grazing

## References

[1] Lelieveld J, Crutzen PJ, Dentener FJ (1998). Changing concentration, lifetime and climate forcing of atmospheric methane. Tellus B.

[2] IPCC. Climate Change 2007: The Physical Science Basis. Contribution of working group I to the fourth assessment report of the Intergovernmental Panel on Climate Change. Cambridge University Press, Cambridge, United Kingdom and New York; 2007.

[3] Aronson EL, Helliker BR (2010). Methane flux in non-wetland soils in response to nitrogen addition: a meta-analysis. Ecology.

[4] Wang Y, Chen H, Zhu Q, Peng C, Wu N, Yang G, Zhu D, Tian J, Tian L, Kang X (2014). Soil methane uptake by grasslands and forests in China. Soil Biol Biochem.

[5] Wang Z, Song Y, Gulledge J, Yu Q, Liu H, Han X (2009). China’s grazed temperate grasslands are a net source of atmospheric methane. Atmos Environ.

[6] Kinney CA, Mosier AR, Ferrer I, Furlong ET, Mandernack KW. Effects of the herbicides prosulfuron and metolachlor on fluxes of CO_2_, N_2_O, and CH_4_ in a fertilized Colorado grassland soil. J Geophys Res Atmos 2004;109(D5). 10.1029/2003JD003656.

[7] Mosier AR, Pendall E, Morgan JA (2003). Effect of water addition and nitrogen fertilization on the fluxes of CH_4_, CO_2_, NOx, and N_2_O following 5 years of elevated CO_2_ in the Colorado Shortgrass Steppe. Atmos Chem Phys.

[8] Mosier AR, Delgado JA (1997). Methane and nitrous oxide fluxes in grasslands in western Puerto Rico. Chemosphere.

[9] Del Grosso SJ, Parton WJ, Mosier AR, Ojima DS, Potter CS, Borken W, Brumme R, Butterbach Bahl K, Crill PM, Dobbie K (2000). General CH_4_ oxidation model and comparisons of CH_4_ oxidation in natural and managed systems. Global Biogeochem Cycles.

[10] Soussana JF, Allard V, Pilegaard K, Ambus P, Amman C, Campbell C, Ceschia E, Clifton-Brown J, Czobel S, Domingues R (2007). Full accounting of the greenhouse gas (CO_2_, N_2_O, CH_4_) budget of nine European grassland sites. Agr Ecosyst Environ.

[11] van den Pol-van Dasselaar A, van Beusichem ML, Oenema O (1999). Effects of nitrogen input and grazing on methane fluxes of extensively and intensively managed grasslands in the Netherlands. Biol Fert Soils.

[12] Pol-van Dasselaar AVD, Beusichem MLV, Oenema O (1997). Effects of grassland management on the emission of methane from intensively managed grasslands on peat soil. Plant Soil.

[13] Wang YS, Xue M, Zheng XH, Ji BM, Du R, Wang YF (2005). Effects of environmental factors on N_2_O emission from and CH_4_ uptake by the typical grasslands in the Inner Mongolia. Chemosphere.

[14] Holst J, Liu C, Yao Z, Brueggemann N, Zheng X, Giese M, Butterbach-Bahl K (2008). Fluxes of nitrous oxide, methane and carbon dioxide during freezing-thawing cycles in an Inner Mongolian steppe. Plant Soil.

[15] Chen W, Wolf B, Yao Z, Bruggemann N, Butterbach-Bahl K, Liu C, Han S, Han X, Zheng X (2010). Annual methane uptake by typical semiarid steppe in Inner Mongolia. J Geophys Res Part D Atmos.

[16] Wei D, Xu-Ri, Tenzin-Tarchen, Wang Y, Wang Y (2015). Considerable methane uptake by alpine grasslands despite the cold climate: in situ measurements on the central Tibetan Plateau, 2008–2013. Global Change Biol.

[17] Tang S, Wang C, Wilkes A, Zhou P, Jiang Y, Han G, Zhao M, Huang D, Schoenbach P (2013). Contribution of grazing to soil atmosphere CH_4_ exchange during the growing season in a continental steppe. Atmos Environ.

[18] Wang C, Tang S, Wilkes A, Jiang Y, Han G, Huang D (2012). Effect of stocking rate on soil-atmosphere CH_4_ flux during spring freeze-thaw cycles in a northern desert steppe, China. PLoS ONE.

[19] Wang C, Han G, Wang S, Zhai X, Brown J, Havstad KM, Ma X, Wilkes A, Zhao M, Tang S (2014). Sound management may sequester methane in grazed rangeland ecosystems. Sci Rep UK.

[20] Chen W, Wolf B, Zheng X, Yao Z, Butterbach-Bahl K, Brueggemann N, Liu C, Han S, Han X (2011). Annual methane uptake by temperate semiarid steppes as regulated by stocking rates, aboveground plant biomass and topsoil air permeability. Global Change Biol.

[21] Bai Y, Wu J, Pan Q, Huang J, Wang Q, Li F, Buyantuyev A, Han X (2007). Positive linear relationship between productivity and diversity: evidence from the Eurasian Steppe. J Appl Ecol.

[22] Yan L, Zhou G, Zhang F (2013). Effects of different grazing intensities on grassland production in China: a meta-analysis. PLOS ONE.

[23] Liu C, Holst J, Brüggemann N, Butterbach-Bahl K, Yao Z, Yue J, Han S, Han X, Krümmelbein J, Horn R (2007). Winter-grazing reduces methane uptake by soils of a typical semi-arid steppe in Inner Mongolia. China Atmos Environ.

[24] Qi Y, Dong Y, Yang X, Geng Y, Liu L, Li M (2005). Effects of grazing on carbon dioxide and methane fluxes in typical temperate grassland in Inner Mongolia, China. Resour Sci.

[25] Praeg N, Wagner AO, Illmer P (2017). Plant species, temperature, and bedrock affect net methane flux out of grassland and forest soils. Plant Soil.

[26] Zhou X, Wang Y, Huang X, Hao Y, Tian J, Wang J (2008). Effects of grazing by sheep on the structure of methane-oxidizing bacterial community of steppe soil. Soil Biol Biochem.

[27] Krogh L, Noergaard A, Hermansen M, Greve MH, Balstroem T, Breuning-Madsen H (2003). Preliminary estimates of contemporary soil organic carbon stocks in Denmark using multiple datasets and four scaling-up methods. Agr Ecosyst Environ.

[28] Lin X, Wang S, Ma X, Xu G, Luo C, Li Y, Jiang G, Xie Z (2009). Fluxes of CO_2_, CH_4_, and N_2_O in an alpine meadow affected by yak excreta on the Qinghai-Tibetan plateau during summer grazing periods. Soil Biol Biochem.

[29] Wang X, Huang D, Zhang Y, Chen W, Wang C, Yang X, Luo W (2013). Dynamic changes of CH_4_ and CO_2_ emission from grazing sheep urine and dung patches in typical steppe. Atmos Environ.

[30] Wan SQ, Hui DF, Luo YQ (2001). Fire effects on nitrogen pools and dynamics in terrestrial ecosystems: a meta-analysis. Ecol Appl.

[31] Lin D, Xia J, Wan S (2010). Climate warming and biomass accumulation of terrestrial plants: a meta-analysis. New Phytol.

[32] Luo YQ, Hui DF, Zhang DQ (2006). Elevated CO_2_ stimulates net accumulations of carbon and nitrogen in land ecosystems: a meta-analysis. Ecology.

[33] Gurevitch J, Hedges LV, Scheiner SM, Gurevitch J (2001). Meta-analysis: combining the results of independent experiments. Design and analysis of ecological experiments.

