# Supplementary material for: Effect of grazing on methane uptake from Eurasian steppe of China
Source: BMC Ecol. 2018 Mar 20;18:11. doi: 10.1186/s12898-018-0168-x (PMC5859401; doi:10.1186/s12898-018-0168-x)
Supplement: Supplementary file 1 — Additional file 1: Note S1. List of studies used in the meta-analysis. [file 12898_2018_168_MOESM1_ESM.docx]

**Effect of grazing on methane uptake from Eurasian steppe of China**

Shiming Tang^1^, Yujuan Zhang^2,^*^✝^, Xiajie Zhai^1^, Andreas Wilkes^3^, Chengjie Wang^4^ & Kun Wang^1,^*^✝^

*Correspondence: [zyj0113@163.com](mailto:zyj0113@163.com); [wangkun@cau.edu.cn](mailto:wangkun@cau.edu.cn)

^✝^Equal contributors

**^1^** Department of Grassland Science, China Agricultural University, Beijing 100193, China, **^2^** Institute of Grassland Science, Chinese Academy of Agricultural Science, Hohhot 010010, China, **^3^** Values for Development Limited, Bury St Edmunds, IP33 3EQ, UK, **^4^** College of Ecology and Environmental Science, Inner Mongolia Agricultural University, Hohhot 010018, China

**Notes S1** A list of studies used in the meta-analysis.

1. Weiwei Chen, Wolf B, Zhisheng Y, Bruggemann N, Butterbach-Bahl K, et al. (2010) Annual methane uptake by typical semiarid steppe in Inner Mongolia. Journal of Geophysical Research - Part D - Atmospheres **115**: D15108-D15110.

2. Chen W, Wolf B, Zheng X, Yao Z, Butterbach-Bahl K, et al. (2011) Annual methane uptake by temperate semiarid steppes as regulated by stocking rates, aboveground plant biomass and topsoil air permeability. Global Change Biology **17**: 2803-2816.

3. Tang S, Wang C, Wilkes A, Zhou P, Jiang Y, et al. (2013) Contribution of grazing to soil atmosphere CH4 exchange during the growing season in a continental steppe. pp. 170-176.

4. Wang C, Tang S, Wilkes A, Jiang Y, Han G, et al. (2012) Effect of Stocking Rate on Soil-Atmosphere CH4 Flux during Spring Freeze-Thaw Cycles in a Northern Desert Steppe, China.

5. Liu C, Holst J, Brüggemann N, Butterbach-Bahl K, Yao Z, et al. (2007) Winter-grazing reduces methane uptake by soils of a typical semi-arid steppe in Inner Mongolia, China. Atmospheric Environment **41**: 5948-5958.

6. Du R, Chen GX, Lv DR, Wang GC (1997) The Primary Research on in situ Measurements of N_2_O and CH_4_ Fluxes from the Inner Mongolia Grassland Ecosystem Climatic and Environmental Research **2** 67-75. (in Chinese)

7. Wang YF, Ji BM, Chen ZZ, Dennis O (2000) Preliminary results of a study on CH_4_ flux in Xilin river basin steppe under different grazing intensities. Acta P hytoecolog ica Sinic: **24** 693-696. (in Chinese)

8. Qi YC, Dong YS, Yang XH, Geng YB, Liu LX, et al. (2005) Effects of Grazing on Carbon Dioxide and Methane Fluxes in Typical Temperate Grassland in Inner Mongolia, China Resources Science **27** 103-109. (in Chinese)

9. Wang YS, Ji BM, Huang Y, Hu YQ, Wang YF (2001) Effects of Grazing and Cultivating on Emission of Nitrous Oxide, Carbon Dioxide and Uptake of Methane from Grasslands. Environmental Science: **22** 7-13. (in Chinese)

10. Wan YF, Li YE, Gao QZ, Duan MJ, Danjiu LB, et al. (2010) Effect of summer grazing intensity on GHG emission in the North Tibet steppe. Pratacultual Science: **27** 1-6. (in Chinese)

11. He GX, Li KH, Song W, Gong YM, Liu XJ, et al. The fluxes of carbon dioxide，methane and nitrous oxide in alpine grassland of the Tianshan Mountains，Xinjiang. Acta Ecologica Sinica: **34** 674-681. (2014) (in Chinese)

12. Wang YS, Hu YQ, Ji BM, Liu GR, Xue M (2002) Research of grazing effects on greenhouse gas emission in Inner Mongolian grasslands. China Environmental Science **22**: 490-494. (in Chinese)

13. Dong YS, Zhang S, Qi YC, Chen ZZ, Geng YB (2000) Variation of soil CO_2_, N_2_O, CH_4_ fluxes in a typical grassland in Inner Mongolia. Chinese science bulletin: **45** 318-322. (in Chinese)

14. Wang YS, Hu YQ, Ji BM, Liu GR, Xue M (2003) study on relationship between the variations of greenhouse gases efflux/uptake and the key environmental factors in Mongolia semi-arid grassland. Advances in Atmospheric Sciences: 119-127. (in Chinese)

15. Li YE, Qin XB, Li WF, Lin ED, Gao QZ. et al. Impacts of no grazing in summer on greenhouse gas emissions from Kobresia humilis alpine meadow. Transactions of the CSAE: **23** 206-211. (2007) (in Chinese)

16. Wang YS, Xue M, Huang Y, Liu GR, Wang MX, et al. (2003) Greenhouse gases emission or uptake in Inner Mongolia natural and free-grazing grasslands. Chinese Journal of Applied Ecology: **14** 372-376. (in Chinese)

17. Zhou P, Han GD, Wang CJ, Liu RX, Jiang YY, et al. (2011) Effects of stocking rates on carbon flux in the desert grassland ecological system of Inner Mongolia. Journal of Inner Mongolia Agricultural University: **32** 59-64. (in Chinese)

18. Wei D, Xu-Ri, Wang Y, Wang Y, Liu Y, et al. (2012) Responses of CO2, CH4 and N2O fluxes to livestock exclosure in an alpine steppe on the Tibetan Plateau, China. Plant Soil **359**: 45-55.

19. Wolf B, Chen W, Brueggemann N, Zheng X, Pumpanen J, et al. (2011) Applicability of the soil gradient method for estimating soil-atmosphere CO2, CH4, and N2O fluxes for steppe soils in Inner Mongolia. Journal of Plant Nutrition and soil science **174**: 359-372.

20. Zhou X, Hao Y (2010) Effect of 5 years long cessation of grazing on methane uptake in a semi-arid grassland. Polish Journal of Ecology **58**: 801-804.

21. Holst J, Liu C, Yao Z, Brueggemann N, Zheng X, et al. (2008) Fluxes of nitrous oxide, methane and carbon dioxide during freezing-thawing cycles in an Inner Mongolian steppe. Plant Soil **308**: 105-117.

22. Wang YS, Xue M, Zheng XH, Ji BM, Du R, et al. (2005) Effects of environmental factors on N_2_O emission from and CH4 uptake by the typical grasslands in the Inner Mongolia. Chemosphere **58**: 205-215.

23. Wang C, Han G, Wang S, Zhai X, Brown J, et al. (2014) Sound management may sequester methane in grazed rangeland ecosystems. Scientific Reports 4.

24. Wang, X. Y., Zhang, Y. J., Huang, D., Li, Z. G. & Zhang, X. Q., Methane uptake and emissions in a typical steppe grazing system during the grazing season. ATMOS ENVIRON 105 14 (2015).

25. Schoenbach, P. et al., Grazing effects on the greenhouse gas balance of a temperate steppe ecosystem. NUTR CYCL AGROECOSYS 93 357 (2012).

26. Rong, Y. P., Ma, L. & Johnson, D. A., Methane uptake by four land-use types in the agro-pastoral region of northern China. ATMOS ENVIRON 116 12 (2015).

27. Lin, X. et al., Experimental Warming Increases Seasonal Methane Uptake in an Alpine Meadow on the Tibetan Plateau. ECOSYSTEMS 18 274 (2015).
